# Comparative Metabolomics Reveals Phosphine-Induced Metabolic Disruptions in *Planococcus citri* (Risso)

**DOI:** 10.3390/ijms26168020

**Published:** 2025-08-19

**Authors:** Junbeom Lee, Soo-Jung Suh, Bong-Su Kim, Dae-Weon Lee

**Affiliations:** 1 Metabolomics Research Center for Functional Materials, Kyungsung University, Busan 48434, Republic of Korea; 2 Plant Quarantine Technology Center, Animal and Plant Quarantine Agency, Gimcheon 39660, Republic of Korea; suhsj97@korea.kr; 3 Department of SmartBio, Kyungsung University, Busan 48434, Republic of Korea

**Keywords:** *Planococcus citri*, mealybug, phosphine (PH_3_), metabolomics, lipidomics, energy metabolism

## Abstract

Phosphine (PH_3_) is a fumigant often used to control insect pests, but its metabolic effects on insect physiology remain unclear. In this study, a comparative metabolomics analysis was performed to elucidate the physiological metabolic pathways affected by PH_3_ exposure in *Planococcus citri*, and significant changes in the metabolic profiles induced by PH_3_ treatment were identified. Principal component analysis and correlation analysis revealed different metabolic changes, and a total of 45 metabolites were identified and mapped to metabolic pathways using the KEGG database. PH_3_ exposure inhibited energy metabolism by down-regulating riboflavin and flavin adenine dinucleotide, which are important cofactors in oxidative phosphorylation and reactive oxygen species generation. In addition, purine and pyrimidine metabolism, essential for nucleotide synthesis and cellular energy homeostasis, were also suppressed. Notably, lipid metabolism was significantly altered, and the juvenile hormone biosynthesis pathway was down-regulated. These results suggest that PH_3_ inhibits electron transport chain activity, induces oxidative stress, and disrupts lipid homeostasis. This study enhances our understanding of the potential biomarkers of PH_3_ exposure, the metabolic processes involved, and the resistance mechanisms that pests may develop in response to such exposure.

## 1. Introduction

Phosphine (PH_3_) is one of the most widely used fumigants for controlling pests in agriculture and plant quarantine. PH_3_ exhibits high efficacy in various insect pests and has excellent penetration due to its high vapor pressure; therefore, it is widely used for purposes such as durable commodities and facility fumigation [1,2,3]. In particular, it is known to have high efficacy in mealybugs, a major pest of imported fresh commodities, and it is expected to be promising as an alternative fumigant to replace methyl bromide, an ozone-depleting substance [4,5,6]. It is commonly commercialized in the form of metal phosphides like aluminum phosphide due to the high fire risk of pure phosphine [7]. In the case of such metal phosphides, ammonia gas is generated as a warning substance, but ammonia gas can cause damage to fruits or vegetables, making it difficult to use on fresh commodities [8]. Recently, a cylinderized product mixed with carbon dioxide has been commercialized, reducing the fire risk. This phosphine product can be applied to fresh commodities as it does not generate ammonia gas [9,10].

The citrus mealybug, *Planococcus citri* (Risso) (Hemiptera: Pseudococcidae), is found across Asia, the Americas, and Europe and is a plant quarantine pest [11]. Nymphs and female adults attach to the stems, branches, and leaves of host plants and suck the sap with their stingers, causing the leaves to wilt, become deformed, turn yellow, and fall early [12]. In addition, they secrete honeydew, which reduces the photosynthetic activity of the leaves, resulting in lower quality fruit, reducing plant vitality, and damaging the appearance of ornamental flowers [13,14,15]. Biological control can be applicable for this pest, including predators, parasites and entomopathogenic fungi [12,16]. Because citrus mealybugs secrete a wax that covers their entire body, agrochemicals that rely on contact toxicity have very little effect on them [17,18]. Therefore, insecticides with a diffusive mechanism are now being considered in toxicity studies, and fumigants have been suggested as an alternative. The effects and mechanism of action of PH_3_ are known in several pests, mainly insects with complete metamorphosis [19,20,21]. However, there are few studies on the metabolic mechanisms of citrus mealybugs, an incomplete metamorphosis insect, exposed to PH_3_ [22]. In this study, the effect of PH_3_ was analyzed based on comparative metabolomics to elucidate the physiological changes in *P. citri*. Additionally, functional relationships among metabolites induced by PH_3_ were demonstrated based on the metabolite–metabolite interaction network.

## 2. Results

### 2.1. Comparative Metabolic Profiling by PH_3_ Exposure

**Figure 1:**
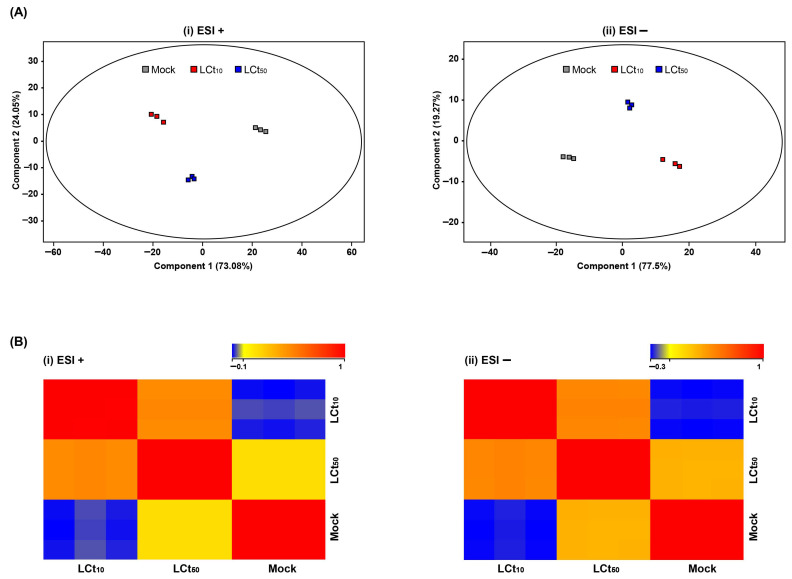
Comparative analysis of metabolic change patterns induced by PH_3_ stress: (**A**) PCA and (**B**) correlation plots of experimental groups with altered metabolites as detected in (**i**) ESI+ and (**ii**) ESI−. Each colored dot indicates *n* = 3 repetitions. The variance percentage includes the *z*-axis values. Colors visually represent the strength and direction of the relationships among variables.

An in vivo assay was performed to investigate the concentration of PH_3_ required to kill *P. citri* (Table 1). The 10% and 50% lethal concentration–time (LCt) values of PH_3_, when administered over 2 h, were 0.161 and 0.216 mg·h/L for adult *P. citri*, respectively. To elucidate the metabolite changes in *P. citri* induced by PH_3_, comparative metabolomics analysis was conducted based on these LCts. When a PCA was performed with raw fold change (FC) data, the reliability of the metabolic analysis was ensured through the use of well-aligned clusters of metabolic data for each group (Figure 1). Significant differences in the positive- (ESI+) and negative-ion modes (ESI−) mass spectrometry were observed after PH_3_ exposure, suggesting that the metabolic physiology of *P. citri* was altered by PH_3_ (Figure 1A). In addition, correlation analysis revealed that there are the relationships between each of the experimental groups. The difference between the control group and LCt_10_ treatment group was clearly discernible (Figure 1B), whereas the correlation among PH_3_ concentrations was low. In the LCt_50_ treatment, a difference was observed more clearly in the ESI+ than in the ESI− (Figure 1B). These results suggest that the metabolome of *P. citri* is changed by PH_3_ exposure and that these altered metabolites can be utilized to determine PH_3_-treatment-specific indicators.

A total of 408 and 216 metabolites were extracted from PH_3_ exposure (both LCt_10_ and LCt_50_) in the ESI+ and ESI− of the mass spectrometry, respectively (Supplementary Data S1), and 90 of these compounds were filtered through annotation processing based on the metabolite database and KEGG ID assignments (Supplementary Data S2). Finally, 45 PH_3_ treatment-specific indicators matching the metabolic pathways of the reference insect, aphid *Acyrthosiphon pisum*, were obtained, along with the peak intensity and related metabolic pathways for each treatment group (Table 2). Among these, 10 and 35 indicators were found to be up- and down-regulated, respectively, suggesting that PH_3_-induced metabolic disruptions occurred in *P. citri*.

### 2.2. Enrichment and Pathway Impact of Altered Metabolites

**Figure 2:**
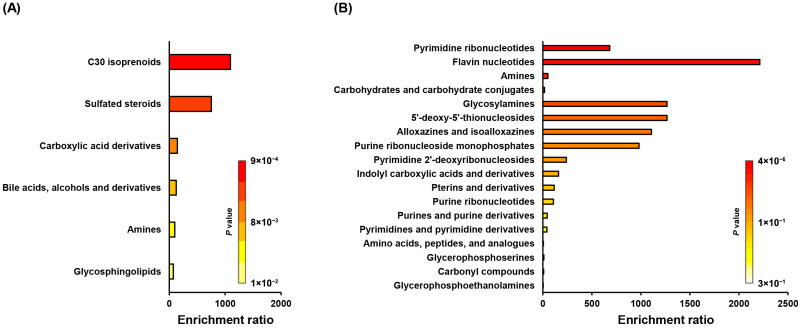
Metabolite set enrichment analysis of altered metabolites. The (**A**) up- and (**B**) down-regulated metabolites were classified into 1250 subchemical metabolite sets based on their chemical structures. The colors of the bar graph describe the *p*-values, with red and orange representing high and low values, respectively.

Metabolite set enrichment analysis (MSEA) identifies biologically meaningful patterns in metabolite concentration changes. Therefore, 45 treatment-specific indicators, categorized as up- or down-regulated by PH_3_ exposure, were classified into metabolite sets based on their chemical structures (Figure 2). Isoprenoids glycosphingolipids, which are involved in membrane structural integrity and cell signaling, were up-regulated, suggesting an adaptive response to PH_3_-induced stress (Figure 2A). In contrast, glycosylamines, glycerophosphoserines, and glycerophosphoethanolamines, key components of membrane phospholipids and glycan-related metabolism, were down-regulated, indicating energy conservation or metabolic suppression (Figure 2B). These results highlight that lipid remodeling is an important response to PH_3_ exposure in *P. citri*. Moreover, purine and pyrimidine derivatives, which are fundamental to nucleic acid biosynthesis and energy metabolism, also exhibited significant down-regulation under PH_3_-induced stress. Potential disruption of nucleotide biosynthesis and energy metabolism could result in impairment of DNA/RNA synthesis or repair mechanisms.

**Figure 3:**
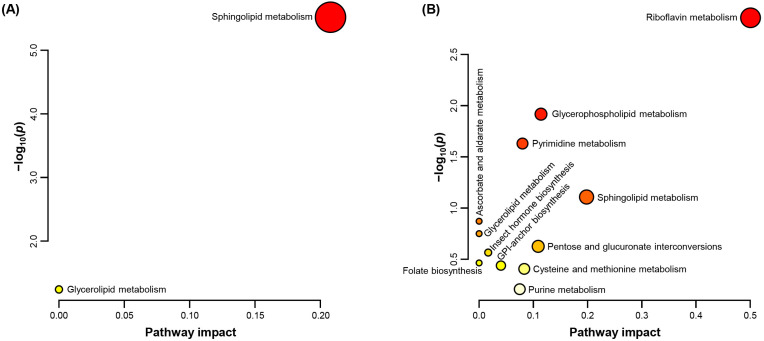
Pathway analysis and impact scores of altered metabolites. The metabolic pathways of metabolites (**A**) up- and (**B**) down-regulated by PH_3_ exposure. Pathway analyses were performed using aphid information (*Acyrthosiphon pisum*, Hemiptera) from the Kyoto Encyclopedia of Genes and Genomes database. The pathway impact was calculated based on the sum of the importance measures of the matched metabolites normalized by the importance of all metabolites in each pathway [28].

To investigate the importance of these 45 indicators within the overall metabolic network of *P. citri*, the metabolites changed by PH_3_ exposure were analyzed using their pathway impact scores from the KEGG database (Figure 3). The results revealed distinct pathway modulations between up-regulated (Figure 3A) and down-regulated (Figure 3B) metabolites. In the up-regulated pathways, sphingolipid metabolism was the most enriched in terms of both statistical significance and pathway impact. Since sphingolipids function to maintain membrane integrity under oxidative stress conditions, membrane remodeling and stress signaling may be enhanced [23]. In contrast, the down-regulated metabolite profiles showed significant inhibition across several biosynthetic and signaling pathways. Notably, riboflavin metabolism was most affected, suggesting a disruption of redox homeostasis in *P. citri*, considering the role of riboflavin-derived cofactors in mitochondrial and oxidative metabolism [24]. In addition, glycerophospholipid metabolism, pyrimidine metabolism, and sphingolipid metabolism were significantly affected, indicating widespread disruption of membrane lipid biosynthesis and nucleotide metabolism [25]. The down-regulation of folate biosynthesis, cysteine and methionine metabolism, and pentose-related metabolism suggests inhibition of cellular biosynthetic capacity and antioxidant defense mechanisms [26,27]. These results suggest that PH_3_ exposure in *P. citri* may act as an energy-conservation or damage-control mechanism by up-regulating stress-adaptive lipid pathways while down-regulating essential biosynthetic pathways.

### 2.3. Metabolite–Metabolite Interaction Network

**Figure 4:**
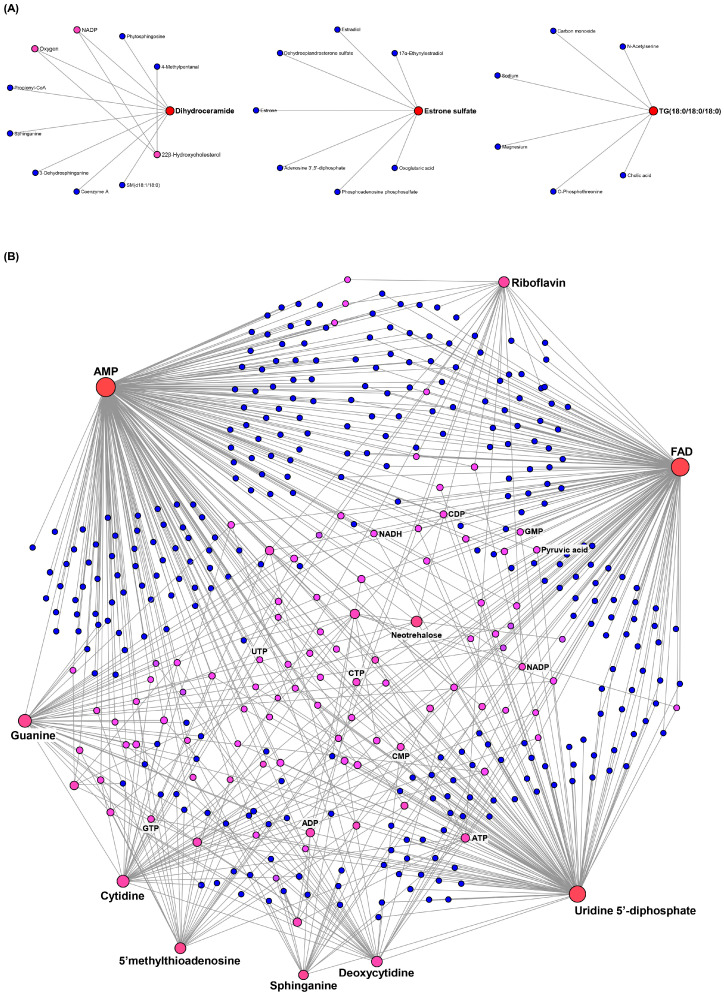
Metabolite–metabolite interaction network for differentially accumulated PH_3_-specific metabolites. The network interaction of metabolites (**A**) up- regulated (subnetwork 1: nodes—11, edges—11, and seeds—2; subnetwork 2: nodes—8, edges—7, and seeds—1; subnetwork 3: nodes—7, edges—6, and seeds—1) and (**B**) down-regulated (nodes—411, edges—737, and seeds—12) by PH_3_ exposure. The metabolic networks are represented as compound networks with metabolites as nodes (circles) and reactions as edges (lines). Nodes indicate correlated metabolites in the network, and lines represent biological relationships between two metabolites. The color of the nodes indicates the betweenness centrality value (metabolites with high betweenness centrality are shown in red, followed by pink and blue), and the size of the nodes represents the degree value (the number of links the node has with other nodes) [29].

Since the metabolite–metabolite network helps to reveal potential functional relationships between metabolites and identify key metabolites, the interaction network of PH_3_-induced metabolites was analyzed (Figure 4). The 10 up-regulated indicators were classified into three subnetworks—dihydroceramide, estrone sulfate, and triradylglycerols—as the central metabolites (Figure 4A and Supplementary Data S3). In addition, metabolic network analysis based on the 35 down-regulated indicators elucidated that metabolites related to purine metabolism (AMP and guanine), pyrimidine metabolism (UDP, cytidine, and deoxycytidine), and riboflavin metabolism (FAD and riboflavin) showed the closest interactions with other metabolites, suggesting that they function as key metabolites within the network (Figure 4B and Supplementary Data S4). These metabolic networks support the results obtained from the MSEA and pathway impact and suggest PH_3_ treatment-specific indicators can be used as potential biomarkers.

### 2.4. Lipid Metabolism Disorders by PH_3_ Stress

Pathway analyses revealed that metabolites involved in sphingolipids (SPs), glycerolipids (GLs) metabolism, and glycosylphosphatidylinositol (GPI) anchor biosynthesis were significantly affected by PH_3_ exposure (Figure 3). Therefore, the changes in lipid profiles induced by PH_3_ stress were investigated. Based on lipid DB annotations, 107 lipids were identified, and differential regulation was evaluated through multivariate statistical analysis (Table 3). PCA and correlation analysis showed that the clusters were well aligned across groups and clearly distinguished from the mock control (Supplementary Data S5). However, there was no significant difference between PH_3_ concentrations, as found in the untargeted metabolic analysis. Lipid types of SPs, GLs, and glycerophospholipids (GPs) were quantitatively changed, but fatty acids (FAs), polyketides (PKs), sterol lipids (STs), or prenols (PRs) were not observed. In addition, most lipids were down-regulated by PH_3_ exposure. These results suggest that lipid metabolism in *P. citri* is disrupted by exposure to PH_3_, regardless of concentration.

## 3. Discussion

Because metabolites immediately reflect physiological responses, metabolomics can effectively reveal changes in the metabolome induced by external stimuli. Although the insecticidal activity of PH_3_ in *P. citri* has been previously studied [30], the physiological metabolic pathways for PH_3_ treatment have not been elucidated. Therefore, metabolic changes induced by PH_3_ exposure in *P. citri* were investigated by comparative metabolomics, and the mechanism of action of PH_3_ was demonstrated. Most of the metabolites changed by PH_3_ affected *P. citri* metabolic pathways related to energy biosynthesis. Notably, its inhibitory effect on the FAD metabolite involved in the ETC cycle can be an important indicator of physiological changes. Additionally, altered levels of the cell membrane lipids glycerophospholipid and sphingolipid demonstrated various effects of PH_3_.

Topological analysis is based on the centrality measure of each metabolite in the metabolic network. Centrality is a comparative measure that indicates the relative position of a particular node to other nodes and is utilized to estimate the relative importance or role of a node in a network configuration [31]. In this study, the network highlights the importance of purine (AMP and guanine) and pyrimidine (cytidine, deoxycytidine, and UDP) metabolites in PH_3_ exposure in *P. citri*, and riboflavin and FAD showed high centrality (Figure 4).

Riboflavin (vitamin B2) is a precursor of flavin adenine mononucleotide (FMN) and flavin dinucleotide (FAD), which are involved in energy metabolism and redox reactions. FMN and FAD act as cofactors for various oxidoreductases and play key roles in glycolysis, the citric acid cycle (TCA cycle), the electron transport chain (ETC), detoxification, and neurotransmission [32]. In cellular respiration in insects, FMN and FAD play roles in accepting and transferring electrons from nicotinamide adenine dinucleotide (NADH) in complex I (NADH dehydrogenase) or producing FADH_2_ form in complex II of the ETC, thereby activating oxidative phosphorylation within the mitochondria and generating adenosine triphosphate (ATP) [33,34]. In addition, FMN and FAD are involved in antioxidant processes that can suppress reactive oxygen species (ROS). Insecticides cause oxidative stress in cells and generate ROS free radicals, and reduced glutathione (GSH) acts as a cofactor to scavenge toxic oxygen radicals [35]. During redox stress, GSH levels decrease, and oxidized glutathione disulfide (GSSG) levels increase by glutathione peroxidase, which functions to prevent lipid peroxidation. On the other hand, in the antioxidant process, FAD transfers hydrogen to activate glutathione reductase (GR), which converts GSSG into GSH [36]. The reduced GSH acts as an endogenous antioxidant within the cell, scavenging ROS. PH_3_ directly interferes with mitochondrial respiration, causing a deficiency in energy metabolism. Briefly, PH_3_ inhibits complex IV (cytochrome c oxidase) of the ETC, which is located in the mitochondrial inner membrane [37,38]. In this study, PH_3_ exposure inhibited riboflavin and FAD metabolites compared to control (Table 2). In previous studies, ethyl formate, a fumigant with similar functional properties to PH_3_, was shown to inhibit FMN, resulting in elevating GSSG [39]. These results support that riboflavin and FAD metabolites are down-regulated in *P. citri* by PH_3_ exposure. Considering the mechanism of action of PH_3_, the inactivation of the ETC, including FAD, may not only inhibit mitochondrial energy metabolism but also promote ROS, causing oxidative stress in *P. citri*.

Ascorbate (AsA) is a hydrophilic antioxidant that neutralizes ROS free radicals, serving as a primary line of defense against oxidative stress in a wide range of organisms, including insects [40,41]. The ascorbate/aldarate metabolic pathway encompasses AsA synthesis, degradation, and recycling, enabling electron transfer from reducing cofactors such as NADPH to remove ROS. This function establishes a direct connection with the GSH-dependent antioxidant detoxification system in insect cells [42,43,44,45]. Recent studies revealed that insecticide-induced ROS accumulation stimulates antioxidant cycles involving both AsA and GSH, with KEGG analysis revealing marked regulation of the ascorbate/aldarate pathway under such stress [46,47]. The AsA-GSH cycle is broadly recognized as a key mechanism for ROS scavenging under environmental challenges [48,49]. In the present study, PH_3_ exposure decreased β-D-glucuronoside, which may reflect either elevated ascorbate accumulation or reduced metabolic activity linked to energy depletion. The alteration in ascorbate/aldarate metabolism observed here suggest enhanced redox imbalance and oxidative stress responses in *P. citri*. Considering the pivotal role of ascorbate in insect antioxidant defense, these metabolic perturbations are likely to exacerbate PH_3_-induced toxicity by impairing detoxification capacity and disrupting redox homeostasis [38,40,50].

The major pathways related to energy metabolism include purine metabolism, which is associated with the production of ATP/GTP, key molecules that control intracellular energy homeostasis and nucleotide synthesis [51]. In addition, pyrimidine nucleotides provide the DNA and RNA compositions needed for cell growth and division [52]. In this study, PH_3_ exposure down-regulated purine (AMP, guanine, and guanosine 3’-phosphate) and pyrimidine (cytidine, deoxycytidine, and UDP) metabolites by 2-fold in *P. citri* (Table 2). These metabolites are involved in the de novo synthesis of purine and pyrimidine and the salvage pathway in insects. A recent study suggested that purine recycling deficiencies cause metabolic and neurobehavioral disturbances in *Drosophila melanogaster* [53].

Comparative proteomic studies on PH_3_ have revealed that cytochrome P450 metabolism-related molecules are significantly up-regulated in PH_3_-resistant insect groups [54,55]. In this study, cytochrome P450 metabolism and four metabolites were inhibited by PH_3_ exposure in *P. citri* (Table 2). These results support the reports that cytochrome P450-related detoxification processes are involved in the acquisition of PH_3_ resistance in insects [54,55].

Because PH_3_-specific metabolites could be utilized as biomarkers, metabolites and their pathways that are specific for PH_3_ exposure were additionally investigated. Interestingly, the metabolism associated with the biosynthesis of juvenile hormone (JH) was completely inhibited by PH_3_ exposure (Table 2 and Figure 3B). Generally, JH biosynthesis and insect metabolism show a positive correlation. In addition, the main JH structure of hemipteran insects is known as the juvenile hormone III skipped bisepoxide (JHSB_3_), which contains an epoxide ring attached to JH III [56]. The process by which JH III is converted to JHSB_3_ is not yet clear. In *P. citri* exposed to PH_3_, the production of JH III diol, degradative form of JH III, was completely inhibited, and JHSB_3_ was not found. This result suggests that JH III diol may be an intermediate in the process of JHSB_3_ production.

A recent study showed that PH_3_-resistant insects had higher levels of lipids (glycerolipids and phospholipids) than susceptible ones [57]. GLs function as a storage for energy shortage [58,59], and cell membrane phospholipids, mainly composed of phosphatidylethanolamine (PE) and GP class, act as a barrier separating the cell from the external environment [60]. Lipids serve as an energy source that enables insects to survive under phosphine-induced stress and provide a suitable environment to protect mitochondria from phosphine. In this study, PH_3_ exposure completely inhibited PE in the glycosylphosphatidylinositol (GPI) anchor biosynthetic pathway (Figure 3B). These results explain the reason most lipids were down-regulated or suppressed in *P. citri*. In addition, cell surface-associated lipids such as GPs and SPs were significantly down-regulated under PH_3_ stress (Table 3). SPs, as key components of lipid rafts, are involved in cell membrane receptor function and signal transduction, and exhibit strong affinity for the GPI anchors [61,62,63]. GPI-anchors are covalently linked to the carboxyl terminus of proteins and function to attach GPI proteins to the lipid bilayer [64,65]. Interestingly, diglycerides (DGs) were up-regulated, whereas triglycerides (TGs) were mostly suppressed (Table 3). This lipid regulation pattern is similar to that of *Drosophila suzukii* after PH_3_ treatment [66]. In insects, lipids are the major component of the fat body and are stored as TG, a form that can be used during long-term energy demands [58,67]. In contrast, diglycerides (DGs), the major lipid in insect hemolymph, are rapidly utilized during short-term energy demands such as flight [58,68]. Considering the mechanism of action of PH_3_ involving energy depletion in mitochondria, the increase in DG and decrease in TG in response to stress suggest that PH_3_ adversely affects the energy metabolic pathway in *P. citri*.

## 4. Materials and Methods

### 4.1. Insect Rearing

The citrus mealybugs, *P. citri*, used in this study were collected from nursery trees in greenhouses (Suwon, Republic of Korea) in 2022. They were mounted on microscope slides for identification. After verifying their identity, the individuals were continuously reared on potato tubers (*Solanum tuberosum* L.), which were provided twice a month, at 25°C with a 60% relative humidity and photoperiod of 16:8 h (light:dark) at the Plant Quarantine Technology Center, Animal and Plant Quarantine Agency (Gimcheon, Republic of Korea) [30].

### 4.2. Phosphine and Thermal Treatment

PH_3_ (Vivakill^®^, 2% PH_3_ + 98% CO_2_) was purchased from FarmHannong (Seoul, Republic of Korea). Adult mealybugs (a mixture of male and female adults) were placed on potato slices in separate 100 mm × 40 mm breeding dishes for each experimental treatment group (SPL, Pocheon, Republic of Korea). PH_3_ was administered for 2 h at 20°C in a 12 L desiccator (DWK Life Sciences, Mainz, Germany). The LCt values of fumigant on *P. citri* were calculated using a Probit analysis [69]. Adults from each group were transferred to glass vials and rapidly frozen in liquid nitrogen to prevent metabolic changes. All treatments and controls were performed in triplicate (*n* = 10).

### 4.3. Metabolite Extraction

Total metabolites were extracted from whole body of *P. citri* adults in triplicate (10 insects/replicate). Briefly, each sample was suspended in 1 mL of extraction solution (3:1:1, methanol/chloroform/water, *v*/*v*/*v*) and homogenized using a Beadbug microtube homogenizer (Benchmark Scientific Inc., Sayreville, NJ, USA) for 3 min. Samples were incubated at room temperature for 20 min and then centrifuged at 20,000× *g* and 4°C for 5 min. The supernatant was filtered through a 0.22 μm pore mesh (Ultrafree-MC, Millipore, Burlington, MA, USA) and immediately loaded into the liquid chromatograph triple-quadrupole time-of-flight mass spectrometry (LC-QTOF/MS; Agilent Technologies 1290 and 6545 System, Agilent Technologies, Santa Clara, CA, USA; Metabolomics Research Center for Functional Materials, Kyungsung University) for metabolomics. The metabolite recovery rate of the sample was investigated with internal standards (L-alanine, Sigma-Aldrich, Oakville, ON, Canada), and the extraction process demonstrated a recovery rate of 50% or greater [66].

### 4.4. Lipid Extraction

Total lipids were extracted from whole body of *P. citri* adults in triplicate (10 insects/replicate) using the modified Bligh and Dyer method, as described previously [70]. Briefly, each sample was suspended in 3 mL of solution (methanol/chloroform, 2:1, *v*/*v*) and homogenized using glass beads. Samples were incubated at room temperature for 20 min and centrifuged at 20,000× *g* for 5 min at 4°C. Supernatants were transferred to new tubes to remove tissue debris. One milliliter of chloroform and 1.8 mL of water were added to each sample, followed by vortexing for 1 min. The lower layer was separated by centrifugation at 2000× *g* for 10 min at 4°C, followed by transfer to a new tube and drying under pure N_2_ gas. Dried samples were then suspended in 200 μL of solution (methanol/chloroform, 1:1, *v*/*v*) and sonicated for 5 min. The resulting supernatants were filtered through 0.22 μm pore size filters (Millipore) and immediately loaded into the LC-QTOF/MS (Agilent Technologies) instrument for lipid analysis. The recovery rates of lipid standards (SPLASH^®^ LIPIDOMIX^®^ Mass Spec Standard, Avanti Research, London, UK) were used to confirm the efficiency of lipid extraction, and the extraction process demonstrated more than 50% recovery rates.

### 4.5. Metabolomics

Untargeted metabolomics was performed using an LC-QTOF/MS instrument (Agilent Technologies) with an electrospray ionization (ESI) source. Since metabolites have ionization preferences depending on their structure and properties, mass spectrometry was performed in both ESI+ and ESI−. For the metabolome analysis, 3 μL of each sample was injected into an InfinityLab Poroshell 120 HILIC-Z column (2.1 mm × 100 mm, 2.7 μm; Agilent Technologies), which was kept at 25°C in its ESI+ and 50°C in its ESI−. The binary mobile phase system utilized in its ESI+, i.e., phase A, was 10 mM of ammonium formate in water containing 0.1% formic acid, while phase B was 10 mM ammonium formate in 90% acetonitrile containing 0.1% formic acid. The mobile phase had a flow rate of 0.25 mL/min and was created under the following conditions: initiation at 2% A, followed by a linear gradient to 2% A over 3 min, 30% A at 11 min, 40% A at 12 min, 95% A at 16 min, 95% A at 18 min, 2% A at 19 min, 2% A at 20 min, and 2% B at 24 min. The binary mobile phase system utilized in ESI−, phase A, was 10 mM of ammonium acetate in water (pH 9.0), while phase B was 10 mM of ammonium acetate in 85% acetonitrile (pH 9.0). The mobile phase had a flow rate of 0.25 mL/min and was created under the following conditions: initiation at 4% A, followed by a linear gradient to 4% A over 2 min, then 12% A at 5.5 min, 12% A at 8.5 min, 14% A at 9 min, 14% A at 14 min, 18% A at 17 min, 35% A at 23 min, 35% A at 24 min, 4% A at 24.5 min, and 4% B at 29 min. The capillary voltage was set to 3.0 kV in the column’s ESI+ and 3.5 kV in its ESI−. Metabolites with a mass within the range of 50 to 1600 *m*/*z* were detected using a quadrupole time-of-flight instrument.

### 4.6. Data Processing and Statistical Analysis

To ensure that the parameters were applied to all samples consistently, the data were analyzed in one batch and normalized using the total ion intensity. All potential metabolites were extracted from the LC peaks of each sample and analyzed using Mass Hunter Qualitative software (Ver. 10.0, Agilent Technologies). These compounds were annotated using the METLIN metabolite database and then filtered, scaled, integrated, and statistically analyzed and visualized using Mass Profiler Professional software (Ver. 14.0; Agilent Technologies). Significant differences between experimental groups were confirmed through a principal component analysis (PCA) and Pearson correlation analysis. Differentially up- or down-regulated metabolites were compared to the mock control group and are defined as changes in entities with [raw fold change (FC)] values > 2 and a *p* < 0.05. Metabolite and lipid data were evaluated using MetaboAnalyst 6.0, https://www.metaboanalyst.ca (accessed on 20 July 2025), and related pathways were visualized using the Kyoto Encyclopedia of Genes and Genomes (KEGG) database.

## Figures and Tables

**Table 1:** Lethal concentration–time of PH_3_ for adult *P. citri* (treatment conditions: 2 h treatment at 20 °C in a 12 L desiccator).

Stage	Number Treated	LCt_10_ (95% CI)	LCt_50_ (95% CI)	Slope ± SE	*df*	*χ* ^2^
Adult	450	0.161 (0.137–0.174)	0.216 (0.205–0.232)	5.17 ± 1.93	13	17.03

**Table 2:** Metabolites found to be differentially expressed under PH_3_-stress (N.D. means not detected).

KEGG ID	Compound	Fold Change (vs. [Mock])		Related Pathway	
		[LCt_10_]	[LCt_50_]		
C00422	TG(12:0/12:0/12:0)	8.765	5.578	api00561	Glycerolipid metabolism
C02960	Cer(d18:1/18:0)	12.796	3.470	api00600	Sphingolipid metabolism
C00319	Sphingosine	1.709	N.D.	api00600	Sphingolipid metabolism
C06126	Galabiosylceramide (d18:1/22:0)	339,388	N.D.	api00600	Sphingolipid metabolism
C12126	Dihydroceramide	7,290,115	N.D.	api00600	Sphingolipid metabolism
C03997	5-Hydroxymethyldeoxycytidylate	213,974	N.D.	api00240	Pyrimidine metabolism
C02538	Estrone 3-sulfate	56,747	N.D.	api01100	Metabolic pathways
C05502	22R-hydroxycholesterol	N.D.	129,099	api01100	Metabolic pathways
C08972	Quillaic acid	N.D.	242,641	api01100	Metabolic pathways
C11472	D-glycero-D-manno-heptose 1,7-bisphosphate	N.D.	115,917	api01250	Biosynthesis of nucleotide sugars
C00350	PE(17:2(9Z,12Z)/20:1(11Z))	−3.167	−1.173	api00563	GPI-anchor biosynthesis
				api00564	Glycerophospholipid metabolism
C00319	D-erythro-sphingosine C-17	−2.251	−1.868	api00600	Sphingolipid metabolism
C00836	C17 sphinganine	−2.648	−2.127	api00600	Sphingolipid metabolism
C03033	Epinephrine glucuronide	−3.985	−1.869	api00040	Pentose and glucuronate interconversions
				api00053	Ascorbate and aldarate metabolism
C00255	Riboflavin (vitamin B2)	−3.677	−1.833	api00740	Riboflavin metabolism
				api02010	ABC transporters
C00242	Guanine	−3.125	−1.762	api00230	Purine metabolism
C00881	Deoxycytidine	−8.308	−2.426	api00240	Pyrimidine metabolism
				api02010	ABC transporters
C16582	2-Hydroxyfelbamate	−3.179	−1.727	api00982	Drug metabolism: cytochrome P450
C14876	S-(2-Hydroxyethyl)-N-acetyl-L-cysteine	−6.718	−2.825	api00980	Metabolism of xenobiotics by cytochrome P450
C00475	Cytidine	−7.167	−2.400	api00240	Pyrimidine metabolism
				api02010	ABC transporters
C06193	Guanosine 3’-phosphate	−3.831	−2.315	api00230	Purine metabolism
C00331	Indolepyruvate	−3.874	−1.644	api00380	Tryptophan metabolism
C02237	5-Oxo-D-proline	−4.788	−2.178	api01100	Metabolic pathways
C00020	Adenosine 5’-monophosphate (AMP)	−3.620	−1.854	api00230	Purine metabolism
C02989	L-Methionine S-oxide	−5.030	−2.178	api00270	Cysteine and methionine metabolism
C06156	D-Glucosamine 1-phosphate	−4.036	−1.826	api01250	Biosynthesis of nucleotide sugars
C00015	Uridine diphosphate (UDP)	−2.803	−1.853	api00240	Pyrimidine metabolism
C00570	CDP-ethanolamine	−3.028	−1.651	api00564	Glycerophospholipid metabolism
C10556	Deoxypodophyllotoxin	−10.196	−2.280	api01100	Metabolic pathways
C02737	PS(18:0/0:0)	−4.339	−3.272	api00564	Glycerophospholipid metabolism
C06542	Ajmaline	−6.059	−1.892	api01100	Metabolic pathways
C19564	4-(Nitrosoamino)-1-(3-pyridinyl)-1-butanone	−2.978	−1.793	api00980	Metabolism of xenobiotics by cytochrome P450
C11680	Cathenamine	N.D.	N.D.	api01100	Metabolic pathways
C00422	TG(16:1(9Z)/14:0/16:1(9Z))[iso3]	N.D.	N.D.	api00561	Glycerolipid metabolism
C16692	Mannopine	N.D.	N.D.	api02010	ABC transporters
C01152	3-Methyl-L-histidine	N.D.	N.D.	api00340	Histidine metabolism
C16505	(10S)-Juvenile hormone III diol	N.D.	N.D.	api00981	Insect hormone biosynthesis
C04874	7,8-Dihydroneopterin	N.D.	N.D.	api00790	Folate biosynthesis
C19606	NNAL-N-glucuronide	N.D.	N.D.	api00980	Metabolism of xenobiotics by cytochrome P450
C00350	PE(12:0/19:1(9Z))	N.D.	−1.260	api00563	GPI-anchor biosynthesis
				api00564	Glycerophospholipid metabolism
C00350	PE(19:1(9Z)/0:0)	N.D.	1.079	api00563	GPI-anchor biosynthesis
				api00564	Glycerophospholipid metabolism
C16365	5-Acetylamino-6-formylamino-3-methyluracil	N.D.	−1.010	api00232	Caffeine metabolism
C00016	Flavin adenine dinucleotide (FAD)	N.D.	−1.875	api00740	Riboflavin metabolism
				api04977	Vitamin digestion and absorption
C00170	5’-Deoxy-5’-(methylthio)adenosine	−4.264	N.D.	api00270	Cysteine and methionine metabolism

**Table 3:** Lipidomic profiling altered by PH_3_ exposure.

Log_2_ Fold Change (vs. [Mock])		LMP ID	Category	Main Class	Compound
[LCt_10_]	[LCt_50_]				
−16.17	−16.17	LMSP02010018	Sphingolipids [SP]	Ceramides [SP02]	Cer(d18:0/13:0)
−1.03	−0.87	LMSP02020015	Sphingolipids [SP]	Ceramides [SP02]	Cer(d18:0/18:1(9Z))
−22.06	−0.38	LMSP02010004	Sphingolipids [SP]	Ceramides [SP02]	Cer(d18:1/16:0)
−18.80	−0.54	LMSP02010006	Sphingolipids [SP]	Ceramides [SP02]	Cer(d18:1/18:0)
−1.20	−1.06	LMSP02010003	Sphingolipids [SP]	Ceramides [SP02]	Cer(d18:1/18:1(9Z))
0.00	15.00	LMSP02010007	Sphingolipids [SP]	Ceramides [SP02]	Cer(d18:1/20:0)
−1.36	−1.48	LMSP02010008	Sphingolipids [SP]	Ceramides [SP02]	Cer(d18:1/22:0)
−1.16	−1.11	LMSP02010026	Sphingolipids [SP]	Ceramides [SP02]	Cer(d18:2/20:0)
−1.39	−1.04	LMSP02010027	Sphingolipids [SP]	Ceramides [SP02]	Cer(d18:2/20:1)
−17.13	−17.13	LMSP02050007	Sphingolipids [SP]	Ceramides [SP02]	CerP(d18:1/24:1(15Z))
2.29	2.10	LMSP0501AA31	Sphingolipids [SP]	Neutral glycosphingolipids [SP05]	GlcCer(d16:1/23:0)
1.76	1.73	LMSP0501AA32	Sphingolipids [SP]	Neutral glycosphingolipids [SP05]	GlcCer(d18:1/23:0)
1.97	1.50	LMSP0501AA10	Sphingolipids [SP]	Neutral glycosphingolipids [SP05]	GlcCer(d18:1/26:1(17Z))
1.17	0.77	LMGL02010095	Glycerolipids [GL]	Diradylglycerols [GL02]	DG(16:1(9Z)/21:0/0:0)[iso2]
18.29	18.12	LMGL02010034	Glycerolipids [GL]	Diradylglycerols [GL02]	DG(17:0/18:2(9Z,12Z)/0:0)[iso2]
1.25	0.87	LMGL02010068	Glycerolipids [GL]	Diradylglycerols [GL02]	DG(17:0/20:2(11Z,14Z)/0:0)[iso2]
1.46	1.22	LMGL02010088	Glycerolipids [GL]	Diradylglycerols [GL02]	DG(18:3(9Z,12Z,15Z)/19:0/0:0)[iso2]
1.16	0.23	LMGL02010190	Glycerolipids [GL]	Diradylglycerols [GL02]	DG(19:0/22:0/0:0)[iso2]
0.00	19.84	LMGL02010250	Glycerolipids [GL]	Diradylglycerols [GL02]	DG(19:0/22:6(4Z,7Z,10Z,13Z,16Z,19Z)/0:0)[iso2]
16.28	16.15	LMGL02010296	Glycerolipids [GL]	Diradylglycerols [GL02]	DG(22:3(10Z,13Z,16Z)/22:5(7Z,10Z,13Z,16Z,19Z)/0:0)[iso2]
−18.49	−18.49	LMGL03010017	Glycerolipids [GL]	Triradylglycerols [GL03]	TG(16:0/16:0/16:1(9Z))[iso3]
−0.70	−1.06	LMGL03010970	Glycerolipids [GL]	Triradylglycerols [GL03]	TG(16:0/20:2(11Z,14Z)/22:1(13Z))[iso6]
−15.83	−15.83	LMGL03010303	Glycerolipids [GL]	Triradylglycerols [GL03]	TG(16:1(9Z)/18:2(9Z,12Z)/20:1(11Z))[iso6]
−16.51	−16.51	LMGL03010948	Glycerolipids [GL]	Triradylglycerols [GL03]	TG(16:1(9Z)/20:4(5Z,8Z,11Z,14Z)/20:5(5Z,8Z,11Z,14Z,17Z))[iso6]
−16.53	−16.53	LMGL03010177	Glycerolipids [GL]	Triradylglycerols [GL03]	TG(17:0/17:1(9Z)/20:0)[iso6]
2.87	2.20	LMGL03010554	Glycerolipids [GL]	Triradylglycerols [GL03]	TG(17:0/18:3(9Z,12Z,15Z)/20:4(5Z,8Z,11Z,14Z))[iso6]
15.02	14.48	LMGL03010196	Glycerolipids [GL]	Triradylglycerols [GL03]	TG(17:2(9Z,12Z)/17:2(9Z,12Z)/18:3(9Z,12Z,15Z))[iso3]
2.03	1.42	LMGL03011824	Glycerolipids [GL]	Triradylglycerols [GL03]	TG(17:2(9Z,12Z)/20:5(5Z,8Z,11Z,14Z,17Z)/22:5(7Z,10Z,13Z,16Z,19Z))[iso6]
1.76	2.06	LMGL03010748	Glycerolipids [GL]	Triradylglycerols [GL03]	TG(18:1(9Z)/18:1(9Z)/20:5(5Z,8Z,11Z,14Z,17Z))[iso3]
17.21	17.33	LMGL03011321	Glycerolipids [GL]	Triradylglycerols [GL03]	TG(18:1(9Z)/20:4(5Z,8Z,11Z,14Z)/20:5(5Z,8Z,11Z,14Z,17Z))[iso6]
−18.36	−18.36	LMGL03011398	Glycerolipids [GL]	Triradylglycerols [GL03]	TG(18:2(9Z,12Z)/20:4(5Z,8Z,11Z,14Z)/20:5(5Z,8Z,11Z,14Z,17Z))[iso6]
2.05	1.56	LMGL03011018	Glycerolipids [GL]	Triradylglycerols [GL03]	TG(18:3(9Z,12Z,15Z)/19:0/20:4(5Z,8Z,11Z,14Z))[iso6]
−14.22	−14.22	LMGL03012401	Glycerolipids [GL]	Triradylglycerols [GL03]	TG(18:3(9Z,12Z,15Z)/22:4(7Z,10Z,13Z,16Z)/22:6(4Z,7Z,10Z,13Z,16Z,19Z))[iso6]
−18.42	−18.42	LMGL03011840	Glycerolipids [GL]	Triradylglycerols [GL03]	TG(20:4(5Z,8Z,11Z,14Z)/20:4(5Z,8Z,11Z,14Z)/20:4(5Z,8Z,11Z,14Z))
−17.36	−17.36	LMGP02010101	Glycerophospholipids [GP]	Glycerophosphoethanolamines [GP02]	PE(10:0/10:0)
16.43	15.77	LMGP02010232	Glycerophospholipids [GP]	Glycerophosphoethanolamines [GP02]	PE(11:0/14:0)[U]
−22.56	−22.56	LMGP02010230	Glycerophospholipids [GP]	Glycerophosphoethanolamines [GP02]	PE(11:0/16:0)[U]
−21.89	−21.89	LMGP02010371	Glycerophospholipids [GP]	Glycerophosphoethanolamines [GP02]	PE(12:0/19:1(9Z))
−21.95	−21.95	LMGP02010397	Glycerophospholipids [GP]	Glycerophosphoethanolamines [GP02]	PE(13:0/20:2(11Z,14Z))
−22.61	−22.61	LMGP02010062	Glycerophospholipids [GP]	Glycerophosphoethanolamines [GP02]	PE(14:0/15:0)[U]
0.00	21.68	LMGP02010419	Glycerophospholipids [GP]	Glycerophosphoethanolamines [GP02]	PE(14:0/22:1(11Z))
−16.58	−16.58	LMGP02010430	Glycerophospholipids [GP]	Glycerophosphoethanolamines [GP02]	PE(14:1(9Z)/17:1(9Z))
22.52	0.00	LMGP02010441	Glycerophospholipids [GP]	Glycerophosphoethanolamines [GP02]	PE(14:1(9Z)/20:1(11Z))
0.58	−20.36	LMGP02010456	Glycerophospholipids [GP]	Glycerophosphoethanolamines [GP02]	PE(15:0/17:1(9Z))
−23.50	−23.50	LMGP02010458	Glycerophospholipids [GP]	Glycerophosphoethanolamines [GP02]	PE(15:0/18:1(9Z))
−23.79	−23.79	LMGP02010041	Glycerophospholipids [GP]	Glycerophosphoethanolamines [GP02]	PE(16:0/18:3(9Z,12Z,15Z))
−25.08	−25.08	LMGP02010509	Glycerophospholipids [GP]	Glycerophosphoethanolamines [GP02]	PE(16:0/19:1(9Z))
−21.21	−21.21	LMGP02010356	Glycerophospholipids [GP]	Glycerophosphoethanolamines [GP02]	PE(16:1(5Z)/16:1(5Z))
−26.03	−26.03	LMGP02010528	Glycerophospholipids [GP]	Glycerophosphoethanolamines [GP02]	PE(16:1(9Z)/19:1(9Z))
22.34	22.19	LMGP02010531	Glycerophospholipids [GP]	Glycerophosphoethanolamines [GP02]	PE(16:1(9Z)/20:2(11Z,14Z))
−21.36	−21.36	LMGP02010251	Glycerophospholipids [GP]	Glycerophosphoethanolamines [GP02]	PE(17:0/14:0)[U]
0.00	23.89	LMGP02010580	Glycerophospholipids [GP]	Glycerophosphoethanolamines [GP02]	PE(17:1(9Z)/20:1(11Z))
−16.42	−16.42	LMGP02010630	Glycerophospholipids [GP]	Glycerophosphoethanolamines [GP02]	PE(18:0/19:0)
−22.82	−22.82	LMGP02011202	Glycerophospholipids [GP]	Glycerophosphoethanolamines [GP02]	PE(18:0/20:3(8Z,11Z,14Z))
−16.27	−16.27	LMGP20020004	Glycerophospholipids [GP]	Glycerophosphoethanolamines [GP02]	PE(18:0/22:6(4Z,7Z,10Z,12E,16Z,19Z)(14OH))
0.00	19.69	LMGP02010768	Glycerophospholipids [GP]	Glycerophosphoethanolamines [GP02]	PE(18:4(6Z,9Z,12Z,15Z)/22:6(4Z,7Z,10Z,13Z,16Z,19Z))
−19.63	−19.63	LMGP02010779	Glycerophospholipids [GP]	Glycerophosphoethanolamines [GP02]	PE(19:0/18:4(6Z,9Z,12Z,15Z))
−19.78	−19.78	LMGP02010816	Glycerophospholipids [GP]	Glycerophosphoethanolamines [GP02]	PE(19:1(9Z)/20:4(5Z,8Z,11Z,14Z))
−28.16	−28.16	LMGP02010851	Glycerophospholipids [GP]	Glycerophosphoethanolamines [GP02]	PE(20:1(11Z)/17:2(9Z,12Z))
−20.02	−20.02	LMGP02010906	Glycerophospholipids [GP]	Glycerophosphoethanolamines [GP02]	PE(20:3(8Z,11Z,14Z)/15:0)
−18.03	−18.03	LMGP02010955	Glycerophospholipids [GP]	Glycerophosphoethanolamines [GP02]	PE(20:4(5Z,8Z,11Z,14Z)/20:5(5Z,8Z,11Z,14Z,17Z))
−16.70	−16.70	LMGP02011167	Glycerophospholipids [GP]	Glycerophosphoethanolamines [GP02]	PE(21:0/18:0)
15.71	15.54	LMGP02050025	Glycerophospholipids [GP]	Glycerophosphoethanolamines [GP02]	PE(22:0/0:0)
−16.39	−0.16	LMGP02010292	Glycerophospholipids [GP]	Glycerophosphoethanolamines [GP02]	PE(22:0/24:1(15Z))
−18.97	−18.97	LMGP02011083	Glycerophospholipids [GP]	Glycerophosphoethanolamines [GP02]	PE(22:2(13Z,16Z)/18:1(9Z))
19.91	0.00	LMGP02020034	Glycerophospholipids [GP]	Glycerophosphoethanolamines [GP02]	PE(O-16:0/21:0)
20.15	19.95	LMGP02020048	Glycerophospholipids [GP]	Glycerophosphoethanolamines [GP02]	PE(O-18:0/18:2(9Z,12Z))
−17.57	−17.57	LMGP02020070	Glycerophospholipids [GP]	Glycerophosphoethanolamines [GP02]	PE(O-20:0/17:2(9Z,12Z))
−22.94	−22.94	LMGP02010347	Glycerophospholipids [GP]	Glycerophosphoethanolamines [GP02]	PE-NMe(16:0/16:0)[U]
−24.09	−24.09	LMGP02010326	Glycerophospholipids [GP]	Glycerophosphoethanolamines [GP02]	PE-NMe2(18:1(9Z)/18:1(9Z))
15.77	15.40	LMGP03050009	Glycerophospholipids [GP]	Glycerophosphoserines [GP03]	PS(14:0/0:0)
18.94	18.83	LMGP03010921	Glycerophospholipids [GP]	Glycerophosphoserines [GP03]	PS(14:0/22:0)
−24.05	−24.05	LMGP03010135	Glycerophospholipids [GP]	Glycerophosphoserines [GP03]	PS(14:1(9Z)/22:1(11Z))
−21.60	−21.60	LMGP03010238	Glycerophospholipids [GP]	Glycerophosphoserines [GP03]	PS(17:0/19:1(9Z))
15.20	15.06	LMGP03050003	Glycerophospholipids [GP]	Glycerophosphoserines [GP03]	PS(18:0/0:0)[U]
1.00	0.79	LMGP03010961	Glycerophospholipids [GP]	Glycerophosphoserines [GP03]	PS(18:0/20:1(11Z))
−17.84	−17.84	LMGP03010960	Glycerophospholipids [GP]	Glycerophosphoserines [GP03]	PS(18:0/20:2(11Z,14Z))
16.47	16.26	LMGP03050011	Glycerophospholipids [GP]	Glycerophosphoserines [GP03]	PS(18:2(9Z,12Z)/0:0)
−18.20	−18.20	LMGP03010458	Glycerophospholipids [GP]	Glycerophosphoserines [GP03]	PS(19:0/16:1(9Z))
18.98	0.00	LMGP03010868	Glycerophospholipids [GP]	Glycerophosphoserines [GP03]	PS(19:0/19:0)
18.05	17.70	LMGP03010536	Glycerophospholipids [GP]	Glycerophosphoserines [GP03]	PS(20:1(11Z)/17:0)
−19.65	0.30	LMGP03010537	Glycerophospholipids [GP]	Glycerophosphoserines [GP03]	PS(20:1(11Z)/17:1(9Z))
18.39	18.34	LMGP03010614	Glycerophospholipids [GP]	Glycerophosphoserines [GP03]	PS(20:3(8Z,11Z,14Z)/21:0)
−22.02	−22.02	LMGP03010733	Glycerophospholipids [GP]	Glycerophosphoserines [GP03]	PS(22:1(11Z)/17:0)
1.11	0.79	LMGP03010768	Glycerophospholipids [GP]	Glycerophosphoserines [GP03]	PS(22:2(13Z,16Z)/18:1(9Z))
−15.14	−15.14	LMGP03010840	Glycerophospholipids [GP]	Glycerophosphoserines [GP03]	PS(22:6(4Z,7Z,10Z,13Z,16Z,19Z)/20:5(5Z,8Z,11Z,14Z,17Z))
−14.47	−14.47	LMGP03030030	Glycerophospholipids [GP]	Glycerophosphoserines [GP03]	PS(P-18:0/14:0)
−15.69	−15.69	LMGP03030053	Glycerophospholipids [GP]	Glycerophosphoserines [GP03]	PS(P-18:0/22:1(11Z))
−18.04	−0.57	LMGP04010123	Glycerophospholipids [GP]	Glycerophosphoglycerols [GP04]	PG(14:1(9Z)/18:3(9Z,12Z,15Z))
1.29	0.85	LMGP04010256	Glycerophospholipids [GP]	Glycerophosphoglycerols [GP04]	PG(17:1(9Z)/17:0)
0.00	15.39	LMGP04010004	Glycerophospholipids [GP]	Glycerophosphoglycerols [GP04]	PG(21:0/22:6(4Z,7Z,10Z,13Z,16Z,19Z))
−15.58	−15.58	LMGP04020088	Glycerophospholipids [GP]	Glycerophosphoglycerols [GP04]	PG(O-16:0/22:6(4Z,7Z,10Z,13Z,16Z,19Z))
−15.83	−15.83	LMGP04020037	Glycerophospholipids [GP]	Glycerophosphoglycerols [GP04]	PG(O-18:0/21:0)
−20.74	−20.74	LMGP04060001	Glycerophospholipids [GP]	Glycerophosphoglycerols [GP04]	PG(O-20:0/0:0)
16.64	15.98	LMGP04020065	Glycerophospholipids [GP]	Glycerophosphoglycerols [GP04]	PG(O-20:0/21:0)
−18.54	−18.54	LMGP06010167	Glycerophospholipids [GP]	Glycerophosphoinositols [GP06]	PI(16:0/22:1(11Z))
−17.98	−17.98	LMGP06010168	Glycerophospholipids [GP]	Glycerophosphoinositols [GP06]	PI(16:0/22:2(13Z,16Z))
0.00	22.05	LMGP06010185	Glycerophospholipids [GP]	Glycerophosphoinositols [GP06]	PI(16:1(9Z)/20:1(11Z))
0.39	−22.33	LMGP06010283	Glycerophospholipids [GP]	Glycerophosphoinositols [GP06]	PI(18:0/18:3(6Z,9Z,12Z))
−21.93	−21.93	LMGP06010428	Glycerophospholipids [GP]	Glycerophosphoinositols [GP06]	PI(19:0/17:1(9Z))
−18.01	−18.01	LMGP06010611	Glycerophospholipids [GP]	Glycerophosphoinositols [GP06]	PI(20:4(5Z,8Z,11Z,14Z)/21:0)
−15.77	−15.77	LMGP10010921	Glycerophospholipids [GP]	Glycerophosphates [GP10]	PA(14:1(9Z)/14:1(9Z))
−16.64	−16.64	LMGP10050024	Glycerophospholipids [GP]	Glycerophosphates [GP10]	PA(18:4(6Z,9Z,12Z,15Z)/0:0)
−17.23	2.22	LMGP10010853	Glycerophospholipids [GP]	Glycerophosphates [GP10]	PA(21:0/13:0)
16.05	15.57	LMGP10010004	Glycerophospholipids [GP]	Glycerophosphates [GP10]	PA(21:0/22:6(4Z,7Z,10Z,13Z,16Z,19Z))
−17.53	−17.53	LMGP10020015	Glycerophospholipids [GP]	Glycerophosphates [GP10]	PA(O-16:0/21:0)
−15.26	−15.26	LMGP10020043	Glycerophospholipids [GP]	Glycerophosphates [GP10]	PA(O-20:0/13:0)

## Data Availability

All data generated or analyzed during this study are included in this published article [and its Supplementary Materials Files].

## Supplementary Materials

The following supporting information can be downloaded at: https://www.mdpi.com/article/10.3390/ijms26168020/s1.
